# Training Practices Among Spanish Natural Elite Bodybuilders in the Pre-Contest Phase

**DOI:** 10.3390/sports14010020

**Published:** 2026-01-05

**Authors:** Eneko Baz-Valle, Sergio Martínez-Gómez, Javier Gene-Morales, Pablo Jiménez-Martínez, Carlos Alix-Fages, Jordan Santos-Concejero

**Affiliations:** 1 Department of Physical Education and Sport, University of the Basque Country UPV/EHU, 01006 Vitoria-Gasteiz, Spain; 2 Department of Health Research, ICEN Research Center, 38002 Santa Cruz de Tenerife, Spain; info@sergiomcoach.com (S.M.-G.);; 3 Research Group in Prevention and Health in Exercise and Sport (PHES), Department of Physical Education and Sports, University of Valencia, 46010 Valencia, Spain; javier.gene@uv.es; 4 Applied Biomechanics and Sport Technology Research Group, Autonomous University of Madrid, 28049 Madrid, Spain

**Keywords:** natural bodybuilding, resistance training, training volume, repetition range, hypertrophy, contest preparation, training frequency

## Abstract

Natural bodybuilders optimize their body composition by combining training and nutrition strategies. This study compared the resistance training practices of amateur and professional natural bodybuilders, during the pre-contest phase and in general. Fifty-six drug-tested Spanish bodybuilders (27 amateurs, 29 professionals) completed a 34-item survey assessing training and competing experience, training frequency, exercise selection, weekly sets per muscle group, repetition ranges, and contest-preparation characteristics. Participants trained ≈5 days·week^−1^ (Amateur: 4.74 ± 0.45; Professional: 4.83 ± 0.47) and most muscle groups were trained >1 and <3 times·week^−1^. Weekly direct sets per muscle group averaged ~8–17, with the highest volumes for back (Amateur: mean 15.3; Professional: mean 17.0 sets·week^−1^) and chest (Amateur: 11.8; Professional: 12.7) and the lowest for hamstrings (Amateur: 8.56; Professional: 8.10). The dominant repetition range was 6–10 reps (Amateur: 74.1%; Professional: 89.7%), with 11–15 reps commonly selected as the secondary range. No statistically significant differences were detected between amateurs and professionals for the main training variables, although professionals showed a trend toward more years competing (*p* = 0.078, *d* = 0.49). In conclusion, high-level natural bodybuilders adhere to practices that are generally in line with current scientific recommendations. However, interindividual variability highlights the need for individualized programming.

## 1. Introduction

Natural bodybuilders are drug-tested athletes who compete in physique contests where standardized judging criteria assess muscle mass, body fat levels, symmetry, and stage presentation [1,2]. These athletes undergo strict anti-doping controls before and/or after every competition, including urinalysis, polygraph, and blood analyses, following World Anti-Doping Agency criteria [3,4,5] and those who fail a drug test are sportingly and economically sanctioned.

Natural bodybuilders typically follow highly structured protocols combining precise nutrition strategies such as protein intake timing and distribution, supplement use, resistance and aerobic training, and sleep optimization routines [6,7,8]. Despite common misconceptions regarding extreme dieting practices, natural bodybuilders typically prepare over several months, typically between 4 and 9, aiming to reduce their body fat percentage while preserving as much lean body mass as possible [2,4,9,10]. Since these athletes aim to present their most muscular and leanest physique, characterized by very low levels of body fat on stage, they often follow a prolonged, well-balanced, and highly adherent hypocaloric diet [4].

Although recent literature has provided evidence-based hypertrophy training recommendations for natural bodybuilders [11,12], little is known about the extent to which these guidelines are implemented in real-world practice. Current observations suggest that the training structures commonly reported in this sport appear to be largely consistent with the framework proposed by Helms and colleagues [7]. As such, these athletes usually complete enough resistance training sessions per week (i.e., frequency) to ensure around 10 sets per muscle group, applying submaximal to maximal relative intensities quantified using the repetitions in reserve (RIR) framework, generally ranging from 4 to 0, with most sets performed in the 6–12 repetition range, with general rest intervals of around 2 min, and with exercise selection primarily oriented towards hypertrophy [7,11,13,14]. In theoretical terms, performing a moderate number of repetitions per set, at submaximal to maximal effort levels and combined with moderate-to-high weekly training volumes is generally recognized as the most effective strategy for maximizing skeletal muscle hypertrophy in resistance-trained individuals.

Although these parameters align with general hypertrophy recommendations [7], the specific practices of natural bodybuilders have been rarely documented in peer-reviewed scientific literature [3,4,7,9]. Natural bodybuilding is a discipline uniquely centered on maximizing skeletal muscle development under drug-tested conditions, making it an ideal model for studying evidence-based hypertrophy training in advanced athletes. Spain is also one of the most competitive countries in the international natural bodybuilding circuit, which provides an opportunity to obtain relevant data from a highly accomplished population. Therefore, this study aimed to explore and compare resistance training practices in natural bodybuilding amateur and professional competitors with the primary goal of maximizing muscle hypertrophy during the pre-contest phase.

## 2. Materials and Methods

### 2.1. Participants

Fifty-six natural Spanish bodybuilders participated in the survey (27 amateurs and 29 professionals). Participants were classified according to their competitive status, allowing for subsequent comparisons between groups. Participants competed in Men’s Physique and/or Bodybuilding divisions and had competed between 2022 and 2024. Both categories were included to optimize the recruitment of high-level male athletes. Although these divisions differ in their judging criteria, particularly regarding lower-body muscularity, both share the same physiological goal of maximizing muscle hypertrophy and minimizing body fat. Two athletes in our sample had competed in both divisions, including one professional in each, reflecting the strong overlap in training and nutritional strategies. The main distinction between divisions is often morphological rather than methodological, and combining both groups increased the representativeness and statistical power of the study without compromising its validity. Regarding the competitive level of the professional athletes recruited for this study, most of them had competed internationally, achieving top-3 placements, including four athletes who had won gold medals at the World Natural Bodybuilding Federation (WNBF) world championships. Among the amateurs, all had achieved top-5 placements at national and/or international bodybuilding competitions, with most of them closely approaching professional qualification. All athletes competed under anti-doping regulations of WNBF Spain and/or the Asociación Española de Culturismo Natural (AECN), both of which adhere to the World Anti-Doping Agency (WADA) Code and prohibited-substance list. Drug testing in these federations is implemented in collaboration with Spain’s National Anti-Doping Organization (CELAD), ensuring full compliance with WADA standards. All doping controls are carried out by accredited officers under official custody and chain-of-custody protocols, following legally established timelines for sample collection, transportation, and analysis. In this framework, competitors undergo pre-competition polygraph conducted exclusively by licensed and certified examiners as a complementary prescreening procedure, while biochemical analyses (urine and, for winners, blood) are performed through CELAD supervised laboratories. Specifically, amateur finalists are subject to in-competition urine testing, whereas all athletes seeking professional status are required to pass mandatory urine and blood testing in order to be eligible for professional qualification. Athletes may also be subjected to random out-of-competition testing. These procedures ensure that the sample represents drug-tested natural competitors under internationally recognized WADA-aligned standards. This study was conducted in accordance with the principles of the Declaration of Helsinki [15] and was approved by the local Research Ethics Committee (ref. CEISH 2018/099).

### 2.2. Procedures

A web-based survey (Google Forms; Google LLC, Mountain View, CA, USA) was designed to collect data from participants. The survey consisted of 34 questions and required approximately 5 min to complete. Draft items were generated from prior surveys and literature [16,17], and then assessed for content adequacy by an independent panel of experienced coaches and researchers in natural bodybuilding. Reviewers provided structured feedback on item relevance, representativeness, clarity, and response options. To evaluate respondent comprehension and response processes, we conducted cognitive interviewing during a pilot test with 15 high-level bodybuilders (think-aloud and targeted probes). Feedback from experts and athletes was used to iteratively refine wording, response scales, and item order, yielding the final 34-item instrument administered in the present study.

The final survey link was distributed directly through coaches with notable competitive success between 2022 and 2024. Coaches were explicitly instructed to forward the survey only to their highest-performing amateur and professional athletes, with the inclusion criterion that all participants train at or near muscular failure, ensuring high-intensity training practices. Additional inclusion criteria required participants to be male, over 18 years of age, and active competitors between 2022 and 2024 in either the WNBF or the AECN, both of which maintain strict anti-doping protocols. Participants also had to have achieved at least a top five placement in a national or international competition and to have complete records of their main pre-contest preparation data. The primary exclusion criterion was the use of performance-enhancing substances; however, this was inherently controlled by the inclusion of only athletes competing in federations that conduct rigorous anti-doping testing. The survey remained open from May to July 2025. All participants provided informed consent electronically, and responses were treated with complete confidentiality.

### 2.3. Variables and Instruments

The survey was structured into two main categories of variables. The first category, participant characteristics, included the competitive level (amateur or professional), years of resistance training experience, years of competitive bodybuilding experience, number of competitions entered in the previous year, number of weeks spent dieting for their most recent competition, body weight at the beginning and end of contest preparation, and highest competitive placement achieved. The second category, training variables, covered weekly training frequency (days per week), training frequency per muscle group (pectoralis, back, quadriceps, biceps, triceps, hamstrings, deltoids), average number of exercises performed per muscle group, total weekly sets per muscle group, average number of sets per exercise, and most commonly used repetition ranges (participants chose their primary and secondary repetition ranges from: 1–5, 6–10, 10–15, and >15 repetitions). The questionnaire included a combination of closed-ended dichotomous items (two possible responses), multiple-choice items with predefined options, and a limited number of open-ended questions requesting specific information such as average contest body weight or best competitive placement. This structure ensured standardized yet comprehensive data collection across all variables. To standardize responses, a working set was defined as a set performed with a load and effort intended to stimulate adaptation (warm-up sets excluded). Direct weekly sets were counted when the target muscle was a primary mover in the exercise (e.g., biceps during elbow-flexion exercises; triceps during elbow-extension exercises); indirect contributions from multi-joint movements were not included for arm musculature. Training frequency per muscle group referred to the number of sessions per week in which that muscle group was deliberately trained. Respondents were instructed that sets were typically performed to or near momentary failure, consistent with the study’s inclusion criterion.

### 2.4. Statistical Analyses

All statistical analyses were conducted with IBM SPSS (version 28; IBM Corp, Armonk, NY, USA). After basic data curation and coding, normality was assessed using the Shapiro–Wilk test. Most variables exhibited non-normal distributions. Inferential analyses were performed using independent *t*-tests or Mann–Whitney U tests, as appropriate. Effect sizes were calculated using Cohen’s *d* [18] and interpreted as negligible (<0.20), small (>0.2 and <0.4), moderate (≥0.4 and <0.8), and large (≥0.80). Comparisons of categorical variables were conducted through Pearson’s Chi-Squared test. Effect sizes for these analyses were reported as Phi (φ) for 2 × 2 tables or Cramer’s V for larger tables and interpreted as very strong (>0.25), strong (>0.15), moderate (>0.10), and weak (>0.05) [19]. Significance for all analyses was set at *p* < 0.05, and trends were identified when *p* < 0.130.

## 3. Results

The average age of participants was 28.63 ± 4.85 years, and their experience in competition was 3.73 ± 2.66 years on average. Participant characteristics are presented in Table 1. There were no significant differences between groups for most variables (all *p* ≥ 0.102, *d* ≤ 0.30). However, a trend toward significance with a moderate effect size (*p* = 0.078, *d* = 0.49) was observed for the number of years competing in bodybuilding, with professionals reporting more experience. All data presented in this section compares amateur and professional competitors; the comparisons between bodybuilders and men’s physique competitors are presented in Supplementary Material Table S1.

Table 2 depicts the number of sessions per week, including total sessions and sessions for each muscle group. There were no significant differences between groups for any of the variables (all *p* ≥ 0.133, *d* ≤ 0.40). Most athletes reported training five days a week, with a frequency of two sessions per muscle group.

Table 3 includes the number of exercises per muscle group a week. There were no differences between groups in any of the variables (all *p* ≥ 0.235, *d* ≤ 0.43). Notably, both professionals and amateurs reported the same most frequent values (mode) for triceps (3 exercises/week), biceps (3 exercises/week), and shoulders (4 exercises/week). However, the most frequently reported values differed between groups for chest (amateurs: 5 exercises/week; professionals: 4 exercises/week), back (amateurs: 6 exercises/week; professionals: 8 exercises/week), quads (amateurs: 5 exercises/week; professionals: 4 exercises/week), and hamstrings (amateurs: 4 exercises/week; professionals: 3 exercises/week).

Table 4 shows the number of sets performed per week for each muscle group, as well as the most and second most used repetition ranges. A trend toward significance was observed for amateurs reporting a higher number of sets per exercise (2.89 sets/exercise) compared to professionals (2.59 sets/exercise), with a moderate effect size (*p* = 0.101, *d* = 0.50).

**Figure 1:**
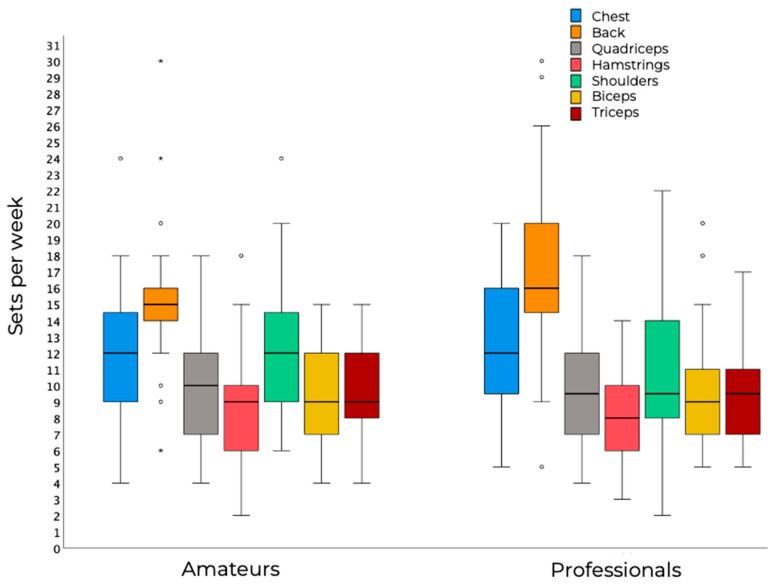
Weekly sets for each muscle group. The boxes represent the interquartile range (IQR) and whiskers 1.5 times the IQR; the black horizontal line inside the boxes represents the median; dots and asterisks represent mild (>1.5 × IQR) and extreme (>3 × IQR) outliers, respectively.

The weekly volume for each muscle group is depicted in Figure 1. Both groups reported the greatest weekly volume for the back, with 95% confidence interval (CI) ranging from 13.58 to 17.09 weekly sets for amateurs and from 14.87 to 19.06 for professionals. Similarly, both groups reported the minimum training volume for the hamstrings, with 95%CI ranging from 7.19 to 9.92 weekly sets in amateurs and 6.96 to 9.25 in professionals.

## 4. Discussion

The present study aimed to investigate resistance training practices among the top natural bodybuilders in Spain. A survey was employed to assess whether current practices align with the training recommendations outlined in the scientific literature. To date, most interventional studies have been conducted in a wide variety of populations, but methodological constraints make it difficult to perform such research with natural bodybuilding competitors. Additionally, we compared amateur and professional competitors to determine whether specific variables distinguish these two populations. To our knowledge, this is the first comparative study examining training practices between amateur and professional natural bodybuilders, although similar comparisons exist in the field of nutrition [9,20]. The main finding was that no differences existed between professional and amateur bodybuilders in competition preparation practices.

Amateur and professional athletes were experienced, with no differences in years of training or competitive experience. However, for years competing, a moderate effect size (*d* = 0.49) favored the professional group, who had on average one additional year of competition experience (4.34 ± 2.84 vs. 3.07 ± 2.32 years). The median difference was even greater (4 vs. 2 years), which may be explained by asymmetry in the distribution of competitive experience in amateur athletes, where a few outliers likely had a longer trajectory than the majority, artificially elevating the group mean. In the study by Chappell, Simper & Helms [4], professional bodybuilders reported more than 12 years of competitive experience versus 2.4 years in amateurs (*p* < 0.001). While the differences in our study were less pronounced, these findings support the notion that prolonged exposure to competition may enhance athletic development [21], potentially by fostering greater motivation and effort toward achieving competitive goals.

The reported contest preparation lengths and weight loss patterns were consistent with established recommendations for preserving lean mass during competition preparation. Amateur athletes reported 28.90 ± 6.26 weeks and professional athletes 27.30 ± 10.60 weeks of preparation, with corresponding weight losses of 14.50 ± 4.59 kg and 11.90 ± 11.30 kg, respectively. These values align with recommendations to lose approximately 0.5–1% of body mass per week over a 3–4-month period [3].

Regarding training frequency, no statistically significant differences were observed between groups for the number of weekly training sessions or muscle-group-specific frequencies, with effect sizes ranging from trivial to small (*d* = 0.03–0.40). On average, athletes trained ~5 days per week (amateurs: 4.74 ± 0.45; professionals: 4.83 ± 0.47) and reported frequencies exceeding 1.5 but below 3 sessions per muscle group per week. These values are consistent with evidence suggesting that frequencies above once per week can be more effective for hypertrophy, while benefits plateau beyond two weekly sessions [22,23].

The average number of weekly exercises per muscle group ranged from 3 to 6, with slightly higher values for the back and chest, and did not differ significantly between amateur and professional athletes (*p* ≥ 0.235). These findings align with prior research showing certain benefits with exercise variation [24,25]. For example, Costa et al. [25] found that performing three different elbow flexion exercises, compared with a single variation, led to greater hypertrophy in the proximal and middle biceps regions, with the multi-exercise group being the only one to significantly increase muscle thickness in the proximal elbow flexors. Such findings suggest that exercise variety may be an important factor for maximizing hypertrophy and for targeting specific muscle regions [26,27].

In terms of training volume, both amateur and professional athletes reported an average of 8–17 weekly sets per muscle group, with the highest volumes assigned to the back and chest, and the lowest to the hamstrings. No statistically significant differences emerged between groups, although effect sizes ranged from trivial to moderate, with professional athletes reporting higher chest and back sets per week. Inter-individual variability was substantial: some athletes reported >25 weekly sets for the back and chest, while in the hamstrings, quadriceps, biceps, and triceps, some reported as few as 4–6 weekly sets. These lower values may be partially explained by Men’s Physique competitors, whose category requirements do not emphasize lower-body development. Conversely, bodybuilders may perform greater training volumes for the lower body, which could partly reduce the relative volume allocated to smaller muscle groups such as the arms. As shown in Supplementary Material Table S1, Men’s Physique competitors in our sample reported fewer training sessions per week for the hamstrings (*p* = 0.004, *d* = 0.85) and more exercises per week for the biceps (*p* = 0.019, *d* = 0.66) and triceps (*p* = 0.021, *d* = 0.63). No other significant differences were found between bodybuilders and Men’s Physique competitors apart from trends of greater training experience in bodybuilders (*p* = 0.053, *d* = 0.60), more training sessions per week (*p* = 0.084, *d* = 0.48), and more shoulder (*p* = 0.068, *d* = 0.59), biceps (*p* = 0.053, *d* = 0.42), and triceps (*p* = 0.062, *d* = 0.43) sets per week for Men’s Physique competitors. In this context, bodybuilding and men’s physique athletes were analyzed together, as the primary aim of the present study was to characterize resistance training practices oriented toward muscle hypertrophy under drug-tested conditions, which constitute a shared framework across both divisions. Notably, the category-specific analyses presented in Supplementary Material Table S1 indicated that, despite some localized differences, the overall pattern of results remained comparable between divisions, suggesting that these differences did not materially affect the main findings.

The present findings are consistent with recent literature indicating that, in trained individuals, optimal hypertrophy may occur with ~12–20 weekly sets per muscle group [12]. This methodological approach, based on quantifying the number of working sets per muscle group, has been supported by previous systematic evidence on training volume quantification and hypertrophy [28]. It is noteworthy that most studies assess the quadriceps, elbow extensors, or elbow flexors, and for the latter two, training volumes often include multi-joint exercise contributions [29]. In our study, biceps and triceps volumes were based solely on direct work, meaning actual weekly volumes, including indirect work, would be higher, potentially falling within the 12–20 set range. While recent quadriceps-focused studies have shown dose–response relationships with progressive volumes up to 52 weekly sets [30,31], these involved single-muscle training protocols. In contrast, Aube et al. [32] reported no significant differences between 12, 18, and 24 weekly sets for quadriceps hypertrophy. When considering fractional sets (0.5 set for agonist muscles in multi-joint exercises), meta-regression analyses have demonstrated a clear dose–response pattern with diminishing returns [33].

No studies to date have directly examined volume–response relationships for back musculature, but given the anatomical complexity (latissimus dorsi, trapezius, rhomboids, teres major/minor, erector spinae, among others), it is plausible that a higher volume is required relative to other muscle groups. For hamstrings, lower reported direct volumes are often justified by the assumption that this muscle group is more susceptible to muscle damage, particularly after exercises with a high eccentric component [34]. However, resistance training-specific evidence is scarce. For example, Maeo et al. [35] found greater hypertrophy with seated leg curl training (longer muscle lengths) versus prone leg curl (shorter lengths) without a corresponding increase in markers of muscle damage. Thus, while the hypothesis of higher susceptibility influencing programming is plausible, the lack of targeted studies in bodybuilders or hypertrophy-focused protocols precludes firm conclusions. Overall, both amateur and professional athletes appear to fall within moderate to moderately high weekly volumes, consistent with Baz-Valle et al. [12] and near the point of greatest efficiency when considering diminishing returns [33].

Additionally, training volume was reported as the average number of weekly sets performed across an entire competitive season. While this provides a general overview of habitual training practices, it is important to acknowledge that volume is not a static variable and fluctuates considerably across different phases of the season. As highlighted by Schoenfeld and Grgic [36], resistance training volume is often periodized, with progressive increases during hypertrophy-oriented phases (off-season) and intentional reductions during maintenance or tapering periods. Consequently, an athlete reporting an average of 13 weekly sets for the pectoralis major may in reality be performing substantially higher volumes during the off-season and considerably lower volumes during contest preparation. Averaging these values across the season may therefore underestimate peak training loads and overestimate the actual volumes performed during energy-restricted phases. This fluctuation is particularly relevant in physique athletes approaching competition, where prolonged or aggressive caloric restriction, low energy availability, and reduced body fat levels have been shown to impair recovery capacity and reduce tolerance to training volume. Case studies and observational data in natural bodybuilders [37,38,39] demonstrate concurrent declines in anabolic hormones (e.g., testosterone, IGF-1), increases in catabolic markers (e.g., cortisol), neuromuscular performance decrements, and heightened perceptions of fatigue in the final weeks before competition. Such physiological and psychological stressors necessitate a reduction in training volume to manage recovery and prevent overtraining. Therefore, the average volumes reported in our questionnaire likely mask these phase-specific adaptations, reflecting neither the highest volumes tolerated in the off-season or “bulking phase” nor the lowest volumes performed during contest preparation.

Regarding repetition ranges, both amateur and professional athletes most frequently reported working in the 6–10 range (74.1% and 89.7%, respectively), followed by 11–15 repetitions (25.9% and 10.3%, respectively). No participants reported 1–5 or >15 repetitions as their primary range. For the second most-used range, distributions were more heterogeneous, with the majority selecting 11–15 repetitions (51.9% in amateurs; 65.5% in professionals), but others reporting 6–10 repetitions (33.3% and 20.7%, respectively) or 1–5 repetitions (14.8% and 13.8%, respectively). No statistically significant differences were found (*p* = 0.128–0.525). Repetition range is inherently linked to load, and current evidence suggests no significant differences in hypertrophy outcomes [40] or in fiber-type–specific hypertrophy [41], if sets are performed to or near failure. In the present study, this criterion was required for inclusion, as the use of RIR in a survey format lacks objectivity without direct observation. Although hypertrophy can be effectively achieved across a wide spectrum of repetition ranges when sets are performed close to failure, competitive bodybuilders tend to prefer moderate or “traditional” hypertrophy ranges (6–15 repetitions), possibly due to cultural and personal preferences, as well as the greater discomfort commonly associated with high-repetition sets performed to failure [42].

This study has several limitations. First, the use of self-reported questionnaires introduces potential recall and perception biases, particularly for variables such as training volume. Additionally, no objective verification of reported training variables was performed, which may limit external validity. Second, while the sample consisted of high-level athletes, their specific backgrounds and competitive contexts restrict the generalizability of findings to the broader bodybuilding population. In this regard, the limited sample size reduced statistical power but increased ecological validity through the inclusion of elite-level participants. Moreover, the absence of female participants represents another limitation, as judging criteria, posing standards, and competitive emphases differ substantially between male and female divisions. Future studies should therefore include female athletes or directly compare male and female natural bodybuilders to explore potential sex-based differences in training practices. Third, for some variables—such as biceps and triceps volume—only direct work was considered, excluding the indirect stimulus from multi-joint exercises targeting other muscle groups. Finally, the cross-sectional design allows for descriptive associations but cannot establish causal relationships between training practices and hypertrophy or performance outcomes. Despite these limitations, the questionnaire underwent content validation by expert judges and pilot testing to ensure clarity and representativeness of the items, which strengthens the reliability of the collected data. Future research should aim to expand the scope of analysis by including international samples and athletes from a broader range of competitive categories and both sexes. Moreover, conducting long-term intervention studies in natural bodybuilders would provide valuable insights into how training variables influence muscle hypertrophy and performance outcomes over time.

## 5. Conclusions

In conclusion, no clear differences in training practices were observed between amateur and professional natural bodybuilders for the variables assessed. Consistent with prior reports, professionals tend to accumulate more years of competitive experience, which may contribute to their competitive status. On average, athletes performed approximately 8–17 direct weekly sets per muscle group. The most frequently used repetition range was 6–10 repetitions, with 11–15 commonly selected as a secondary range. Training frequency per muscle group was typically greater than 1 and less than 3 sessions per muscle group per week, and athletes generally employed multiple exercises to train each muscle group. Taken together, these results indicate that high-level natural bodybuilders apply practices that are broadly consistent with contemporary scientific recommendations, while the observed interindividual variability supports the need for individualized programming. Future longitudinal or intervention studies in elite cohorts could clarify and support the present findings regarding the training variables measured, as well as others not assessed.

## Figures and Tables

**Table 1:** Training experience and outcomes.

Variable	Level		Body Builders		Men’s Physique		Sig.	φ
Category	Amateurs (*n* = 27)		16 (59.3%)		11 (40.7%)		0.102	0.22
	Professionals (*n* = 29)		10 (37%)		17 (63%)			
	Total (*n* = 56) *		26 (46.4%)		28 (50%)			
**Variable**		**Mean**	**SD**	**Median**	**IQR**	**Mode**	**Sig.**	**Cohen’s *d***
Age (years)	Amateurs	28.61	5.66	27.0	4.75	26	0.487	0.06
	Professionals	28.64	4.07	28.5	5	27		
Training experience (years)	Amateurs	7.85	3.71	8	4.5	5	0.163	0.29
	Professionals	8.90	3.61	8	6.0	6		
Competing experience (years)	Amateurs	3.07	2.32	2	2.5	2	0.078	0.49
	Professionals	4.34	2.84	4	3.0	3		
Competitions in the previous year	Amateurs	2.70	1.10	3	1.0	2	0.809	0.15
	Professionals	2.55	0.87	3	1.0	3		
Weeks on diet	Amateurs	28.90	6.26	28.5	6.5	28	0.488	0.19
	Professionals	27.30	10.60	27.0	12.3	20		
Weight change (kg)	Amateurs	−14.50	4.59	−14.0	3.0	−13	0.185	0.30
	Professionals	−11.90	11.30	−13.0	6.5	−13		

Notes for the table: Frequencies, percentages (in parentheses), and descriptive statistics are presented in the upper and lower sections of the table, respectively. * The frequencies do not add up to the total “n” due to two participants, who were competitors of both modalities, Body Building and Men’s Physique. SD: Standard deviation; IQR: Interquartile range; Sig.: *p*-value of statistical significance.

**Table 2:** Training sessions per week.

Variable		Mean	SD	Median	IQR	Mode	Sig.	Cohen’s *d*
Training sessions/week	Amateurs	4.74	0.45	5	0.5	5	0.506	0.19
	Professionals	4.83	0.47	5	0	5		
Chest trainings/week	Amateurs	1.85	0.46	2	0	2	0.645	0.14
	Professionals	1.79	0.41	2	0	2		
Back trainings/week	Amateurs	1.93	0.39	2	0	2	0.140	0.40
	Professionals	1.76	0.44	2	0	2		
Quads trainings/week	Amateurs	1.70	0.47	2	1.0	2	0.516	0.17
	Professionals	1.62	0.49	2	1.0	2		
Biceps trainings/week	Amateurs	1.96	0.34	2	0	2	0.574	0.06
	Professionals	1.93	0.70	2	0	2		
Triceps trainings/week	Amateurs	1.96	0.34	2	0	2	0.559	0.06
	Professionals	1.93	0.65	2	0	2		
Hamstrings trainings/week	Amateurs	1.78	0.51	2	0.5	2	0.385	0.25
	Professionals	1.66	0.48	2	1.0	2		
Shoulder trainings/week	Amateurs	2.22	0.58	2	0	2	0.939	0.03
	Professionals	2.24	0.79	2	1.0	2		

SD: Standard deviation; IQR: Interquartile range; Sig.: *p*-value of statistical significance.

**Table 3:** Number of exercises per muscle group per week.

Variable		Mean	SD	Median	IQR	Mode	Sig.	Cohen’s *d*
Chest exercises/week	Amateurs	4.33	1.47	5	2.0	5	0.235	0.43
	Professionals	4.97	1.48	4	2.0	4		
Back exercises/week	Amateurs	5.70	1.66	6	2.0	6	0.287	0.34
	Professionals	6.24	1.46	6	3.0	8		
Quads exercises/week	Amateurs	4.04	1.60	4	2.5	5	0.913	0.02
	Professionals	4.07	1.41	4	2.0	4		
Hamstrings exercises/week	Amateurs	3.56	1.40	4	1.0	4	0.502	0.17
	Professionals	3.34	1.01	3	1.0	3		
Shoulders exercises/week	Amateurs	4.74	1.77	4	2.0	4	0.822	0.07
	Professionals	4.86	1.92	5	3.0	4		
Biceps exercises/week	Amateurs	3.48	0.94	3	1.0	3	0.467	0.29
	Professionals	3.83	1.39	4	2.0	3		
Triceps exercises/week	Amateurs	3.42	0.90	3	1.0	3	0.317	0.36
	Professionals	3.83	1.31	4	2.0	3		

SD: Standard deviation; IQR: Interquartile range; Sig.: *p*-value of statistical significance.

**Table 4:** Number of sets per muscle group per week.

Variable		Mean	SD	Median	IQR	Mode	Sig.	Cohen’s *d*
Chest sets/week	Amateurs	11.80	4.40	12	5.5	12	0.479	0.22
	Professionals	12.70	4.22	12	6.0	12		
Back sets/week	Amateurs	15.30	4.44	15	2.0	15	0.126	0.33
	Professionals	17.00	5.41	16	5.3	20		
Quads sets/week	Amateurs	10.00	3.69	10	5.0	6	0.980	0.02
	Professionals	9.97	3.59	10	5.0	12		
Hamstrings sets/week	Amateurs	8.56	3.46	9	4.0	9	0.519	0.14
	Professionals	8.10	2.90	8	4.0	8		
Shoulder sets/week	Amateurs	12.00	4.28	12	5.5	12	0.338	0.18
	Professionals	11.20	5.47	10	6.0	9		
Biceps sets/week	Amateurs	9.04	2.94	9	5.0	8	0.845	0.15
	Professionals	9.54	3.82	9	3.5	10		
Triceps sets/week	Amateurs	9.11	2.61	9	4.0	9	0.667	0.17
	Professionals	9.62	3.26	10	4.0	10		
Sets per exercise	Amateurs	2.89	0.70	3	1.0	3	0.101	0.50
	Professionals	2.59	0.50	3	1.0	3		
		**1–5 reps**	**6–10 reps**	**11–15 reps**	**>15 reps**	**Mode**	**Sig.**	**Cramer’s V**
Most used repetition range	Amateurs	0%	74.1%	25.9%	0%	6–10	0.128	0.20
	Professionals	0%	89.7%	10.3%	0%	6–10		
Second most used repetition range	Amateurs	14.8%	33.3%	51.9%	0%	11–15	0.525	0.15
	Professionals	13.8%	20.7%	65.5%	0%	11–15		

SD: Standard deviation; IQR: Interquartile range; Sig.: *p*-value of statistical significance; reps: repetitions.

## Data Availability

All data associated with this study will be made available upon reasonable request to the corresponding author.

## Supplementary Materials

The following supporting information can be downloaded at: https://www.mdpi.com/article/10.3390/sports14010020/s1, Supplementary Material Table S1: Comparisons between bodybuilders and men’s physique competitors.
