# Supplementary material for: Training Practices Among Spanish Natural Elite Bodybuilders in the Pre-Contest Phase
Source: Sports (Basel). 2026 Jan 5;14(1):20. doi: 10.3390/sports14010020 (PMC12846200; doi:10.3390/sports14010020)
Supplement: Supplementary file 1 [file sports-14-00020-s001.zip › sports-3919703-supplementary.pdf]

Supplementary Material S1. Comparisons between bodybuilders and men's physique competitors.

Variable	Category	Mean	Std. Deviation	95% Confidence Interval		Sig.	Cohen's d
				Lower	Upper		
Age	Bodybuilding	29.36	5.04	27.28	31.44	0.274	0.25
	Men's physique	28.11	4.83	26.20	30.02		
Training experience (years)	Bodybuilding	9.50	4.14	7.83	11.17	0.053	0.60
	Men's physique	7.36	2.95	6.21	8.50		
Competing experience (years)	Bodybuilding	4.00	3.11	2.74	5.26	0.813	0.18
	Men's physique	3.50	2.30	2.61	4.39		
Competitions in the previous year	Bodybuilding	2.81	0.98	2.41	3.20	0.239	0.32
	Men's physique	2.50	0.96	2.13	2.87		
Weeks on diet	Bodybuilding	29.72	6.11	27.20	32.24	0.114	0.31
	Men's physique	27.04	10.53	22.87	31.20		
Weight change (kg)	Bodybuilding	-14.02	4.49	-15.83	-12.20	0.835	0.16
	Men's physique	-12.55	11.64	-17.06	-8.03		
Training sessions/week	Bodybuilding	4.65	0.49	4.46	4.85	0.084	0.48
	Men's physique	4.86	0.36	4.72	5.00		
Chest trainings/week	Bodybuilding	1.88	0.33	1.75	2.02	0.226	0.31
	Men's physique	1.75	0.52	1.55	1.95		
Back trainings/week	Bodybuilding	1.92	0.27	1.81	2.03	0.190	0.34
	Men's physique	1.79	0.50	1.59	1.98		
Quads trainings/week	Bodybuilding	1.77	0.43	1.60	1.94	0.127	0.42
	Men's physique	1.57	0.50	1.38	1.77		
Biceps trainings/week	Bodybuilding	2.00	0.57	1.77	2.23	0.763	0.13
	Men's physique	1.93	0.54	1.72	2.14		
Triceps trainings/week	Bodybuilding	1.96	0.53	1.75	2.17	0.786	0.005
	Men's physique	1.96	0.51	1.77	2.16		
Hamstrings trainings/week	Bodybuilding	1.92	0.39	1.76	2.08	0.004	0.85
	Men's physique	1.54	0.51	1.34	1.73		
Shoulder trainings/week	Bodybuilding	2.15	0.54	1.93	2.37	0.698	0.19
	Men's physique	2.29	0.81	1.97	2.60		
Chest exercises/week	Bodybuilding	4.62	1.65	3.95	5.28	0.972	0.005
	Men's physique	4.61	1.37	4.08	5.14		
Back exercises/week	Bodybuilding	5.73	1.76	5.02	6.44	0.358	0.30
	Men's physique	6.21	1.42	5.66	6.77		
Quads exercises/week	Bodybuilding	4.31	1.57	3.67	4.94	0.304	0.28
	Men's physique	3.89	1.42	3.34	4.44		
Hamstrings exercises/week	Bodybuilding	3.62	1.27	3.10	4.13	0.233	0.30
	Men's physique	3.25	1.14	2.81	3.69		
Shoulders exercises/week	Bodybuilding	4.42	1.81	3.69	5.16	0.283	0.32
	Men's physique	5.00	1.81	4.30	5.70		
Biceps exercises/week	Bodybuilding	3.27	1.08	2.83	3.71	0.019	0.66

	Men's physique	4.04	1.23	3.56	4.51		
Triceps exercises/week	Bodybuilding	3.27	1.08	2.83	3.71	0.021	0.63
	Men's physique	3.96	1.13	3.52	4.41		
Chest sets/week	Bodybuilding	11.85	4.16	10.16	13.53	0.626	0.15
	Men's physique	12.46	4.37	10.77	14.16		
Back sets/week	Bodybuilding	15.73	6.08	13.28	18.19	0.181	0.10
	Men's physique	16.22	3.47	14.85	17.59		
Quads sets/week	Bodybuilding	10.58	3.47	9.18	11.98	0.247	0.27
	Men's physique	9.61	3.78	8.14	11.07		
Hamstrings sets/week	Bodybuilding	8.38	2.95	7.19	9.58	0.474	0.09
	Men's physique	8.11	3.37	6.80	9.41		
Shoulder sets/week	Bodybuilding	10.08	4.17	8.39	11.76	0.068	0.59
	Men's physique	12.93	5.33	10.86	15.00		
Biceps sets/week	Bodybuilding	8.62	3.68	7.13	10.10	0.053	0.42
	Men's physique	10.04	3.13	8.80	11.28		
Triceps sets/week	Bodybuilding	8.73	3.23	7.43	10.04	0.062	0.43
	Men's physique	10.00	2.67	8.97	11.03		
Sets per exercise	Bodybuilding	2.85	0.67	2.57	3.12	0.284	0.33
	Men's physique	2.64	0.56	2.43	2.86		
